# Knee Joint Biomechanics in Physiological Conditions and How Pathologies Can Affect It: A Systematic Review

**DOI:** 10.1155/2020/7451683

**Published:** 2020-04-03

**Authors:** Li Zhang, Geng Liu, Bing Han, Zhe Wang, Yuzhou Yan, Jianbing Ma, Pingping Wei

**Affiliations:** ^1^Shaanxi Engineering Laboratory for Transmissions and Controls, Northwestern Polytechnical University, Xi'an 710072, China; ^2^Hong-Hui hospital, Xi'an Jiaotong University College of Medicine, Xi'an 710054, China; ^3^State Key Laboratory for Manufacturing Systems Engineering, Xi'an Jiaotong University, Xi'an 710054, China

## Abstract

The knee joint, as the main lower limb motor joint, is the most vulnerable and susceptible joint. The knee injuries considerably impact the normal living ability and mental health of patients. Understanding the biomechanics of a normal and diseased knee joint is in urgent need for designing knee assistive devices and optimizing a rehabilitation exercise program. In this paper, we systematically searched electronic databases (from 2000 to November 2019) including ScienceDirect, Web of Science, PubMed, Google Scholar, and IEEE/IET Electronic Library for potentially relevant articles. After duplicates were removed and inclusion criteria applied to the titles, abstracts, and full text, 138 articles remained for review. The selected articles were divided into two groups to be analyzed. Firstly, the real movement of a normal knee joint and the normal knee biomechanics of four kinds of daily motions in the sagittal and coronal planes, which include normal walking, running, stair climbing, and sit-to-stand, were discussed and analyzed. Secondly, an overview of the current knowledge on the movement biomechanical effects of common knee musculoskeletal disorders and knee neurological disorders were provided. Finally, a discussion of the existing problems in the current studies and some recommendation for future research were presented. In general, this review reveals that there is no clear assessment about the biomechanics of normal and diseased knee joints at the current state of the art. The biomechanics properties could be significantly affected by knee musculoskeletal or neurological disorders. Deeper understanding of the biomechanics of the normal and diseased knee joint will still be an urgent need in the future.

## 1. Introduction

Since the number of the old and obese worldwide has been increasing yearly, the research on human motion dysfunction is getting more and more attention. The knee joint, as the main lower limb motor joint, is the most vulnerable and susceptible joint [1]. Knee impairments are the common physical problems which impact the normal living ability and mental health of these patients [2]. The influences mainly contain the supporting body weight, the assisting lower limb swing, and the absorbing strike shock [3]. The movement biomechanics, as an important branch of biomechanics, studies the coordination of the bones, muscles, ligament, and tendons in various human movements [4]. The complex interaction of these structures allows the knee to withstand tremendous forces during various normal movements [1]. Therefore, it is an urgent need to study the movement biomechanics of the normal and diseased knee joint for the assistance or rehabilitation of human locomotor function.

In the last decade, several related review papers appeared and could be divided into two aspects, normal knee biomechanics and diseased knee biomechanics. For the former, Masouros et al. [5] analyzed the knee kinematics and mechanic and surrounding soft tissue in detail. The research pointed out that the knowledge of these structures was very useful for the diagnosis and evaluations of treatment. Wang et al. [6] reviewed the modeling and simulation methods of human musculoskeletal systems. The knee kinematics and kinetics in six common motions including walking, jogging, stair ascent, stair descent, squatting, and kneeling were discussed. Chhabra et al. [1] reported the anatomic structures and their relationships in the uninjured knee joint, which provided the critical guidance for the reconstruction of the multiple ligament injured knee joint. Madeti et al. [4] discussed various model formulations of the knee joint, including mathematical, two-dimensional, and three-dimensional models. And the forces acting on the knee joint had also been compared. For the latter, Flandry et al. [7] provided an overview of the surgical anatomy of the knee joint and emphasized connective tissue structures and common injury patterns. Woo et al. [8] reviewed the biological and biomechanical knowledge of normal knee ligaments, as well as the anatomical, biological, and functional perspectives of the current reconstruction knowledge following knee ligament injuries. The research also provided guidance for improving the treatment of knee ligament injuries. Louw et al. [9] assessed the effects of the occluded vision on the knee kinematics and kinetics during functional activities, such as squatting, stepping down, drop landing, hopping, and cutting movements in healthy individuals and the individuals with anterior cruciate ligament injury or reconstruction. Sosdian et al. [10] discussed the effects of knee arthroplasty on the kinematics and kinetic properties of the frontal plane and sagittal plane during the stance phase of normal walking. The results showed that the peak knee adduction angle and moment were decreased, but the peak knee flexion moment was increased after knee arthroplasty. However, to our knowledge, there is no review that synthesized the literature discussing the movement biomechanics of both the normal and the diseased knee joint.

Understanding the knee biomechanics is a prerequisite for designing knee assistive devices and optimizing rehabilitation exercises. This paper provides an overview of the current biomechanical knowledge on normal and injured knee joints. For better assessment of the function of the knee joint, the biomechanical parameters including angle, moment, power, and stiffness from various researchers in different daily motions are reviewed and compared. For better understanding the kinematics and kinetics of real knee movement, the polycentric rotation in the sagittal plane and biomechanics in the coronal plane are also discussed. Further, the common knee disorders including musculoskeletal and neurological disorders and their influences on the knee biomechanics are also reviewed and discussed. We hypothesized that the comprehensive understanding of the knee joint biomechanics in physiological and pathological conditions could significantly improve the design of knee assistive devices and rehabilitation exercise programs.

The rest of this paper is organized as follows. In Section 2, the search strategies adopted for the literature review are provided. In Section 3, the selected literatures including the biomechanical properties of normal knee joint and the knee diseased effects on the biomechanics are summarized. In Section 4, the limitations of the current studies are briefly discussed and the recommendations for future research are provided.

## 2. Methods

This review was conducted in accordance with Preferred Reporting Items for Systematic Reviews and Meta-Analyses (PRISMA) [11]. We systematically searched electronic databases including ScienceDirect, Web of Science, PubMed, Google Scholar, and IEEE/IET Electronic Library for potentially relevant articles. The following terms were used as keywords (identical for all databases): “knee joint,” “gait,” “knee biomechanics,” “knee disease,” and “sports biomechanics.” Given the fast advancement in acquisition equipment and theoretical research of knee biomechanics, the search time range was set from 2000 to November 2019. A total of 1787 articles were retrieved initially. The daily life activities were mainly considered in this review, the articles about more complex activities, such as squats, hops, cut manoeuvres, were excluded. After 679 articles were excluded, 1108 articles about daily life activities were selected. In addition, review of all references cited by the selected articles and more insight into other relevant authors' studies yielded an additional 35 articles for possible inclusion. Then, all selected articles were input into Excel to eliminate duplicates. After 511 duplicates were removed, 632 articles were assessed for inclusion.

**Figure 1:**
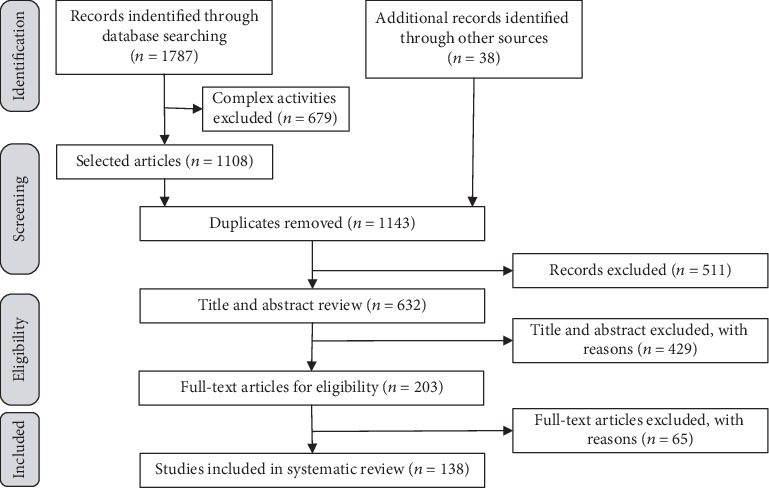
The PRISMA flow diagram of study selection process.

Studies were considered eligible if they met the following inclusion criteria: normal knee kinematics related, normal knee dynamics related, diseased knee kinematics related, diseased knee dynamics related, English, and full-text articles. Two reviewers (LZ and ZW) independently assessed the title and abstracts of the potential studies. After an initial decision, the full text of the studies that potentially met the inclusion criteria were assessed before a final decision was made. A senior reviewer (GL) was consulted in cases involving disagreement. After exclusion of irrelevant titles and screening of abstracts, 203 articles remained. Subsequently, detailed full-text screening based on the inclusion criteria was carried out, and 65 articles were excluded. Finally, 138 full-text articles were examined for full review. The search process is demonstrated using the following diagram shown in Figure 1.

## 3. Results

We divided the 138 selected articles, which fulfilled the literature search inclusion criteria, into two groups: biomechanical properties of normal knee joint and biomechanical properties of diseased knee joint. For the former, the real movement of a normal knee joint and the normal knee biomechanics of four kinds of daily motions in the sagittal and coronal planes, which include normal walking, running, stair climbing, and sit-to-stand, were discussed and analyzed. For the latter, an overview of the current knowledge on the movement biomechanical effects of common knee musculoskeletal disorders (KOA) and knee neurological disorders (SCI, stroke, and CP) were provided.

### 3.1. Biomechanical Properties of Normal Knee Joint

#### 3.1.1. Knee Biomechanics of Daily Motions in Sagittal Plane

Walking, running, stair climbing, and sit-to-stand are very frequent motions in human's daily life. In all of the motions, the main functions of the knee joint include supporting the body weight (BW), absorbing shock of heel strikes, and assisting lower limbs swing [3]. According to the previous researches, the passive knee flexion could reach 160 deg in the sagittal plane [1, 5, 12]. The peak load through the knee joint is 2-3 BW during walking, 2-5 BW during sit-stand-sit, 4-6 BW during stair climbing, and 7-12 BW during running [12–14]. In this section, the ROM, maximum moment, maximum power, and stiffness of the knee joint are mainly discussed because they are the key indexes for the design of knee assistive device and optimization of rehabilitation exercises.

**Figure 2:**
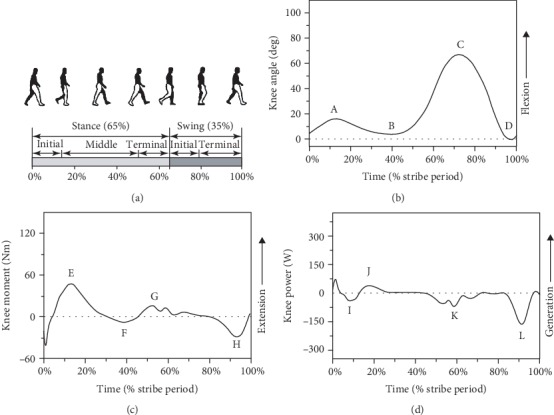
A sketch map of motion and the typical curves of knee angle, moment, and power in the sagittal plane for a walking gait cycle. (a) Sketch map of walking motion [14]. (b) Knee angle-time curve ((A) first peak knee flexion angle, (B) first peak knee extension angle, (C) second peak knee flexion angle, and (D) second peak knee extension angle). (c) Knee moment-time curve ((E) first peak knee extension moment, (F) first peak knee flexion moment, (G) second peak knee extension moment, and (H) second peak knee flexion moment). (d) Knee power-time curve ((I) first peak knee absorption power, (J) first peak knee generation power, (K) second peak knee absorption power, and (L) third peak knee absorption power) [16, 17].

As shown in Figure 2(a), the walking gait can be divided into two main phases: stance (about 0-65% of gait) and swing phases (about 65-100% of gait) [15, 16]. The stance phase consists of three subphases: initial (heel strike to foot flat), middle (foot flat to opposite heel strike), and terminal stance (opposite heel strike to toe off) [16, 17]. The knee joint in the stance phase is regarded as a shock damping mechanism to accept the BW [18]. The swing phase consists of two subphases: initial (toe off to knee maximum flexion) and terminal swing (knee maximum flexion to heel strike) [16, 17]. The main function of the knee in the swing phase is assisting flexion-extension for toe clearance, foot placement, and taking over the load in the next step [19, 20]. Zheng [21] reported that the knee biomechanics is affected mainly by walking speed. With the speed increased, the ROM, maximum extension moment, and maximum absorption power would increase.  Figure 2(b) shows the typical knee angle-time curve. There are two peak flexion (A and C) and extension (B and D) angles. Points A and B occur in the stance phase, and C and D occur in the swing phase. Comparing the two peak flexion angles, the value in the swing phase is always greater than that in the stance phase.  Table 1 gives the values of these points from 18 studies. The ranges of points A, B, C, and D are from 6 to 28 deg, -2 to 5 deg, 53 to 78 deg, and -5 to 16 deg, respectively. In general, the ROM is around 53 to 75 deg for normal walking.  Figure 2(c) shows the typical knee moment-time curve. There are two peak extension (E and G) and flexion (F and H) moments. Point H occurs in the swing phase and the others occur in the stance phase.  Table 2 gives the values of these points from 11 studies. The values of these points vary considerably between different studies. The ranges of points E, F, G, and H are from 0.129 to 0.945 Nm/kg, -0.675 to 0.067 Nm/kg, 0.101 to 0.466 Nm/kg, and -0.420 to 0.086 Nm/kg, respectively. The first peak extension moment is always greater than the second. But it is hard to determine who is bigger between the two peak flexion moments. In general, the range of moment is about 0.458 to 1.265 Nm/kg for normal walking.  Figure 2(d) shows the typical knee power-time curve. It includes one peak generation power (J) and three peak absorption powers (I, K, and L). Point L occurs in the swing phase and the others occur in the stance phase. For the knee joint, there is only absorption power in the swing phase. And in the whole gait cycle, knee absorption powers are much larger than generation powers. Mooney and Herr [22] found that the mean net knee power is about -18 W (mean generation and absorption power is about 18 W and -36 W, respectively).  Table 3 gives the values of these points from 10 studies. The ranges of points I, J, K, and L are from -1.736 to -0.116 W/kg, 0.286 to 0.834 W/kg, -1.935 to -0.403 W/kg, and -2.712 to -0.321 W/kg, respectively. In general, the range of power is about 1.035 to 3.214 W/kg for normal walking.

**Figure 3:**
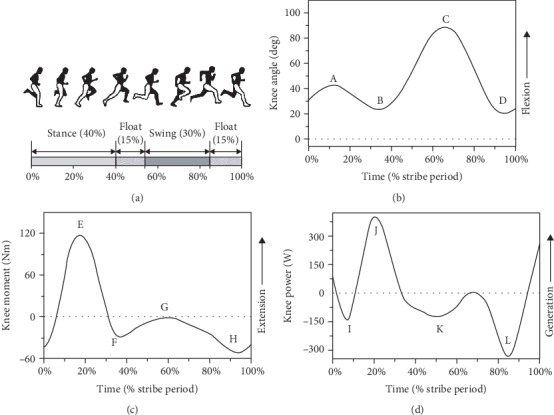
A sketch map of motion and the typical curves of knee angle, moment, and power in the sagittal plane for a running cycle. (a) Sketch map of running motion [14]. (b) Knee angle-time curve ((A) first peak knee flexion angle, (B) first peak knee extension angle, (C) second peak knee flexion angle, and (D) second peak knee extension angle). (c) Knee moment-time curve ((E) first peak knee extension moment, (F) first peak knee flexion moment, (G) second peak knee extension moment, and (H) second peak knee flexion moment). (d) Knee power-time curve ((I) first peak knee absorption power, (J) first peak knee generation power, (K) second peak knee absorption power, and (L) third peak knee absorption power) [23, 122, 123].

As shown in Figure 3(a), the running cycle can be divided into four main phases: stance (heel strike to toe off), first float (toe off to opposite heel strike), swing (opposite heel strike to opposite toe off), and second float phases (opposite toe off to heel strike) [14]. The knee main function in running is similar to that in walking. Comparing Figures 2 and 3, it can be observed that the curves of angle, moment, and power in running are also similar to that in walking. Hamner and Delp [23] reported that the knee biomechanics is mainly affected by running speed. With increasing speed, the ROM, maximum extension moment, and maximum absorption power would increase.  Figure 3(b) shows the typical knee angle-time curve in a gait cycle and Table 4 gives the angles of points A, B, C, and D from 7 studies. The ranges of points A, B, C, and D are from 36 to 60 deg, 13 to 29 deg, 80 to 129 deg, and 10 to 21 deg, respectively. In general, the ROM of the knee joint is around 60 to 115 deg for running.  Figure 3(c) shows the typical knee moment-time curve in a running cycle and Table 5 gives the moments of points E, F, G, and H from 5 studies. The ranges of points E, F, G, and H are from 1.157 to 2.574 Nm/kg, -0.259 to 0.320 Nm/kg, 0.135 to 0.585 Nm/kg, and -1.474 to -0.277 Nm/kg, respectively. The range of moment is about 1.434 to 3.904 Nm/kg for running.  Figure 3(d) shows the typical knee power-time curve in a running cycle and Table 6 gives the powers of points I, J, K, and L from 6 studies. The ranges of points I, J, K, and L are from -1.706 to -12.567 W/kg, 2.739 to 9.405 W/kg, -3.456 to -1.525 W/kg, and -3.456 to -6.732 W/kg, respectively. The range of power is about 8.724 to 21.972 W/kg. This emphasizes that the ranges of knee angle, moment, and power in running are far more than those in normal walking.

**Figure 4:**
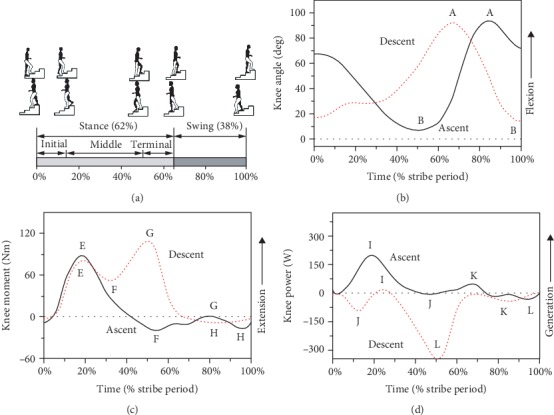
A sketch map of motion and the typical curves of knee angle, moment, and power in sagittal plane for stair ascent and stair descent. (a) Sketch map of the stair ascent and stair descent motion. (b) Knee angle-time curve ((A) peak knee flexion angle and (B) peak knee extension angle. (c) Knee moment-time curve ((E) first peak knee extension moment, (F) first peak knee flexion moment, (G) second peak knee extension moment, and (H) second peak knee flexion moment). (d) Knee power-time curve ((I) first peak knee generation power, (J) first peak knee absorption power, (K) second peak knee generation power, and (L) second peak knee absorption power) [24, 25].

As shown in Figure 4(a), the stair climbing cycle (including stair ascent and descent) can be divided into two main phases: stance phase (about 0-62% of the cycle) and swing phase (about 62-100% of the cycle) [24, 25]. The stance phase consists of three subphases: initial (foot contact to opposite toe off), middle (opposite toe off to opposite foot contact), and terminal stance (opposite foot contact to toe off) [24, 26, 27]. Riener et al. [24] indicated that the knee biomechanics is mainly affected by the rate of leg length and stair height.  Figure 4(b) shows the typical knee angle-time curves in a stair ascent and stair descent cycle. They all include one peak flexion (A) and extension (B) angle. For stair ascent, point A occurs in the swing phase and B occurs in the terminal stance phase. And for stair descent, point A occurs in the terminal stance phase and B occurs in the swing phase.  Table 7 gives the values of these points from 7 studies. The ranges of points A and B are from 83 to 102 deg and 0 to 11 deg for stair ascent and from 83 to 105 deg and 1 to 19 deg for stair descent, respectively. In general, the ROM of the knee joint is around 78 to 94 deg for stair ascent and 76 to 90 deg for stair descent.  Figure 4(c) shows the typical knee moment-time curves in a stair ascent and descent cycle. They all include two peak extension (E and G) and flexion (F and H) moments. For stair ascent, points E and F occur in the stance phase and G and H occur in the swing phase. And for stair descent, points E, F, and G occur in the stance phase and H occurs in the swing phase.  Table 8 gives the values of these points from 7 studies. The ranges of points E, F, G, and H are from 0.454 to 1.409 Nm/kg, -0.556 to -0.145 Nm/kg, 0.027 to 0.144 Nm/kg, and -0.314 to -0.121 Nm/kg for stair ascent and from 0.007 to 1.512 Nm/kg, -0.070 to 0.662 Nm/kg, 0.365 to 1.620 Nm/kg, and -0.266 to 0.040 Nm/kg for stair descent, respectively. In general, the range of moment is about 1.010 to 1.815 Nm/kg for stair ascent and 0.435 to 1.815 Nm/kg for stair descent.  Figure 4(d) shows the typical knee power-time curves in a stair ascent and descent cycle. They all include two peak generation (I and K) and absorption (J and L) powers. For stair ascent, the whole curve lies in the generation area mostly. And for stair descent, the whole curve lies in the absorption area mostly.  Table 9 gives the values of these points from 4 studies. The ranges of points I, J, K, and L are from -1.044 to 2.887 W/kg, -0.228 to 0.071 W/kg, 0.447 to 1.020 W/kg, and -0.739 to -0.265 W/kg for stair ascent and from -0.212 to 0.569 Nm/kg, -3.621 to -0.248 Nm/kg, -1.326 to -0.429, and -5.485 to -2.077 Nm/kg for stair descent, respectively. In general, the range of power is about 1.309 to 3.481 W/kg for stair ascent and 2.114 to 6.054 W/kg for stair descent.

**Figure 5:**
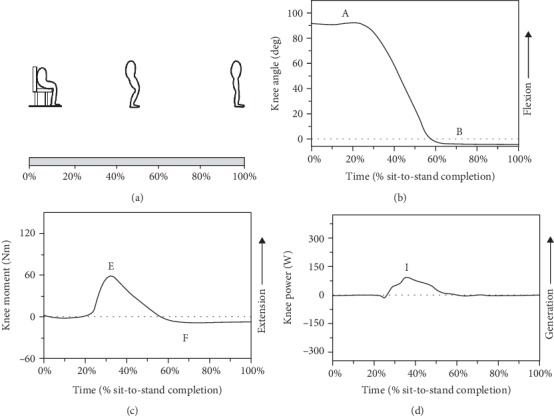
A sketch map of motion and the typical curves of knee angle, moment, and power in the sagittal plane for sit-to-stand. (a) Sketch map of sit-to-stand cycle [28]. (b) Knee angle-time curve ((A) peak knee flexion angle and (B) peak knee extension angle). (c) Knee moment-time curve ((E) peak knee extension moment and (F) peak knee flexion moment). (d) knee power-time curve ((I) peak knee generation power) [37, 124].

As shown in Figure 5(a), the sit-to-stand begins in a sit posture and ends in a stand posture. Figures 5(b)–5(d) show the typical knee angle-time, moment-time, and power-time curves in sit-to-stand cycle, respectively. For the knee joint, there are only extension angle, extension moment, and generation power in the whole sit-to-stand movement. The maximum angle, moment, and power occur in nearly the same time that the buttocks leave the chair. Hurley et al. [28] represented that the biomechanics of knee joint is mainly affected by the rate of leg length and chair height.  Table 10 gives the experimental results of knee angle from 6 studies. The ranges of points A and B are from 82 to 96 deg and -3 to 22 deg, respectively. In general, the ROM of the knee joint is around 60 to 87 deg for sit-to-stand cycle.  Table 11 gives the experimental results of knee moment from 9 studies. The ranges of points E and F are from 0.619 to 2.187 Nm/kg and -0.198 to 0.609 Nm/kg, respectively. In general, the range of moment is about 0.619 to 1.578 Nm/kg for sit-to-stand cycle. The researchers about knee power in sit-to-stand is rare, and only two researchers have been found. Spyropoulos et al. [29] reported that the knee power was about 1.973 W/kg for sit-to-stand. But Kamali et al. [30] pointed out that the value was about 0.560 W/kg for sit-to-stand.

**Figure 6:**
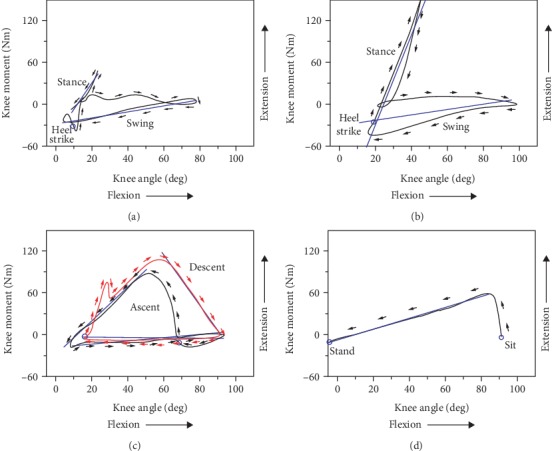
The moment-angle (stiffness) curves of the knee joint for normal walking, running, stair climbing, and sit-to-stand. (a) Normal walking [20, 34]. (b) Running [35, 36]. (c) Stair ascent and stair descent [24]. (d) Sit-to-stand [37].

Because of the complicated interaction of the underlying biological mechanisms, the knee joint demonstrates a spring-like behavior in common motions [31–33].  Figure 6 shows the typical knee moment-angle curves in the sagittal plane. A linear relationship can be seen during the sit-to-stand, and the weight acceptance and swing phase of walking, running, and stair climbing. Quasistiffness refers to the slope of the linear fit to the knee moment-angle curve [33]. During walking, running, and stair climbing, a high stiffness in the weight acceptance phase and a low stiffness in the swing phase can be observed. For walking, Zhu et al. [20] and Wang [12] found that the knee quasistiffness was around 3.0 and 2.27 Nm/deg in the stand phase. Sridar et al. [34] indicated that the knee quasistiffness was around 1.07 Nm/deg in the swing phase. For running, Elliott et al. [35, 36] found that the knee quasistiffness was around 0.38 Nm/deg in the swing phase and 6.6 Nm/deg in the stand phase. For stair climbing, Riener et al. [24] reported that the knee quasistiffness was around 2.37 Nm/deg and 2.42 Nm/deg in the weight acceptance phase of stair ascent and stair descent and 0.19 Nm/deg and 0.04 Nm/deg in the swing phase of stair ascent and stair descent, respectively. For sit-to-stand, Wu et al. [37] reported that the knee quasistiffness was around 1.1 Nm/deg.

#### 3.1.2. The Real Motion and Coronal Plane Biomechanics of Knee Joint

Since the nonuniform shape of the knee articular surface and the complicated physical structure of the femur and tibia, the knee motion cannot be modeled as simple as a perfect hinge [38–40]. The real knee joint moves with a polycentric motion, whereby the center of rotation changes during the rotation [41]. The femur and tibia can be approximated as a bielliptical structure, so the tibia rolls on the femur resulting in anterior-posterior (A-P) translation during the flexion-extension motion [40]. When the rotation angle is less than 20 deg, there would be a small A-P translation. Thus, the movement of a real knee joint can be approximated as pure rolling around the fixed center. But when the rotation angle is more than 20 deg, the A-P translation begins to increase, the amplitude of which can exceed 19 mm. Thus, the knee motion can be approximated as a gradual transition from pure rolling, the coupled motion of rolling, and sliding to pure sliding [38, 40, 42]. Smidt [43] reported that the trajectory of the center of knee seems to be a J-shaped curve in the sagittal plane.

In addition to the motion in the sagittal plane, the knee joint also has internal-external rotation in the horizontal plane [44]. During the last 10-15 deg before complete extension, the medial femoral condyle is internally rotated and the tibia is externally rotated. At the same time, the lateral meniscus is anteriorly translated and the medial meniscus is posteriorly translated. Because of the larger contact surface of the medial tibiofemoral joint, the length of the medial femoral condyle is longer than that of the lateral, and because of the limitations of cruciate-collateral ligaments and quadriceps femoris on knee motion, the knee joint is self-locking as an eccentric wheel to maintain the stability of the joint during the knee extension motion [44, 45]. Blankevoort et al. [46], Churchill et al. [47], and Hollister et al. [48] found that the flexion-extension and internal-external rotation cause the trajectory of the knee center seem to be a spiral curve.

In the coronal plane, the knee adduction moment and the loads of knee medial and lateral compartments are key parameters of biomechanics. For the former, Gaasbeek et al. [49], Russell [50], and Briggs et al. [51] found that the maximum adduction moment is about 0.31, 0.36, and 0.26 Nm/(kg·m) in walking, respectively. Ferber et al. [52], Sinclair [53], and Gehring et al. [54] reported that the maximum adduction moment is about 0.52, 0.53, and 0.58 Nm/(kg·m) in running, respectively. Law [26] and Musselman [27] represented that the maximum adduction moment is about 0.44 and 0.34 Nm/kg in stair climbing, respectively. Trepczynski et al. [55] reported that the maximum adduction moment is about 0.45 Nm/kg in sit-to-stand. For the latter, Russell [50] found that the normal knee joint always had a little varus, in other words, the medial compartment bears more load than the lateral compartment. Specogna et al. [56] reported that the weight-bearing line (WBL) was different in each phase of the gait. Cao [57] reported that the medial compartment bears 60-80% of the load. Pagani et al. [58] found that about 70% joint force pass through the medial compartment to the ground.

### 3.2. Biomechanical Properties of Diseased Knee Joint

According to the pathogeny, the knee disorders can be mainly divided into musculoskeletal and neurological disorders. For the former, the pathogeny is inside the knee joint, but the neural control system of these patients is normal. Knee osteoarthritis (KOA), knee ligament injury, and meniscus injury are the most common forms of these disorders and will be mainly discussed in this section. Some evidences showed that the partial assistance from an external mechanism can alleviate the symptoms [59]. For the latter, the actuator of the knee is normal, but the knee control system or more advanced control system is injured. Although it is not considered a knee joint disease in the medical field, the neurological disorders can influence the knee movement biomechanics. Spinal cord injury (SCI), stroke, and cerebral palsy (CP) are the most common forms of these disorders and will be mainly discussed in this section. Some researchers pointed out that the partial or entire assistance from an external mechanism and rehabilitation training can recover the ambulatory ability of this patients [60, 61].

#### 3.2.1. Knee Musculoskeletal Disorders and Its Biomechanical Effects

KOA, one of the major health problems, affects 7-17% of individuals especially for the elder, obese, and previous limb injury people [62–65]. Nearly 46% of adults will develop painful KOA in at least one knee joint over their lifetime [66]. By 2020, the KOA is predicted to become the fourth leading cause of disability globally [67]. The etiology and progression of KOA are multifactorial, which includes the increasing tibiofemoral force, the femoral shaft curvature changes, enlarging bone marrow lesions, compartment cartilage loss, joint space narrowing, and tibial plateau compression. [63, 68]. From the biomechanical view, these causes will change the tibiofemoral alignment and influence the load distribution, and then result in the deterioration of KOA [69]. Due to the medial compartment bearing about 70% of the total force, KOA is more commonly observed in the medial compartment (MKOA) than the lateral compartment with a ratio of up to 4 times [58, 59].

**Figure 7:**
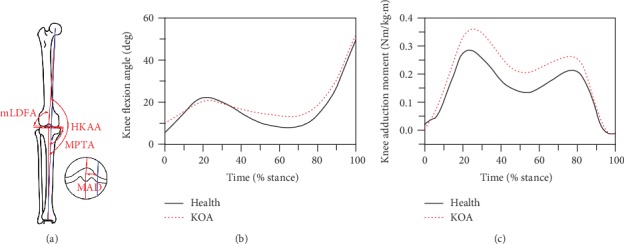
The knee alignment measurement methods and the effect of KOA on flexion angle and adduction moment. (a) Sketch map of HKAA, mLDFA, MPTA, and MAD [61]. (b) Knee flexion angles of health and KOA individuals [48]. (c) Knee adduction moments of health and KOA subjects [48].

Medical radiological assessment, kinematics analysis, kinetics analysis, and knee muscle analysis are the common biomechanical methods for KOA, as shown in Table 12. In the medical radiological assessment aspect, the hip-knee-ankle angle (HKAA) on the full-0limb radiograph is regarded as the gold standard of alignment measurement, as shown in Figure 7(a) [63, 69]. Chao et al. [70] reported that the normal HKAA was about 178.8 deg and the angle is less than the value represented by genu varum. Russell [50] found that the HKAA of normal and MKOA were about 177.7 deg and 174.2 deg, respectively. As shown in Figure 7(a), mechanical-lateral-distal-femoral angle (mLDFA), medial-proximal-tibial angle (MPTA), and joint-line-convergence angle (JLCA) are also commonly used as the measurement parameters [68]. The normal values of these angles are 85-90 deg, 85-90 deg, and 0-2 deg, respectively. The mLDFA greater than 90 deg, MPTA less than 85 deg, or JLCA greater than 2 deg represent genu varum [71]. The mechanical axis deviation (MAD) is another measurement method. The normal MAD is about 8 mm in the medial, and the value greater than the normal MAD represents genu varum [71]. Besides, the WBL ratio and medial or lateral joint space also used to characterize the KOA. Russell [50] pointed out that the WBL ratio, medial joint space, and lateral joint space were about 41.4%, 4.5 mm, and 5.5 mm for normal individuals and 24.2%, 2.8 mm and 7.9 mm for MKOA, respectively. In the knee kinematics aspect, Russell [50] reported that the knee flexion pattern was similar, but the magnitude was lower for MKOA patients compared to that for normal subjects, as shown in Figure 7(b). Zhu et al. [72] found that the KOA patients presented a longer gait time, a smaller stride length and ROM, a greater knee flexion angle at heel strike, and an unobvious fluctuation of knee flexion angle in the stand phase of walking. Alzahrani [73] indicated that the MKOA patients presented slower walking speeds, shorter step lengths, longer stance and double support time, and smaller cadence, stride length, and knee ROM. In the knee kinetics aspect, Russell [50] described that the knee adduction moment pattern was similar, but the magnitude was higher for MKOA patients compared to that for normal subjects in walking, as shown in Figure 7(c). Astephen et al. [74] observed that the knee adduction moment in MKOA patients was greater than that in the normal in mid-stance. Guo et al. [75] found that the MKOA patients possessed a greater peak adduction moment during stair climbing. Rudolph et al. [76] and Schmitt and Rudolph [77] pointed out that the peak knee flexion moment in KOA patients was smaller than that in the normal during early and late stance phases. Fitzgerald et al. [78] reported that a 4-6 deg increase in varus alignment could increase around 70-90% medial compartment load during single limb bearing. Lim et al. [79] indicated that genu varum exceeding 5 deg at baseline was associated with greater functional deterioration over 18 months than the value of 5 deg or less. Kemp et al. [80] observed that a 20% increase in the peak adduction moment could increase the KOA progression risk. In the knee muscle aspect, Slemenda et al. [81], Hurley et al. [82], and Oreilly et al. [83] found that the KOA patients had smaller quadriceps strength and muscle activation. Lim et al. [79] indicated that there was no significant relationship between the varus malalignment and the EMG ratio of VM and VL. Russell [50] reported that the medial muscle (VM-ST and VM-MG) and lateral muscle (VL-BF and VL-LG) cocontraction indices were not significantly different between MKOA patients and normal person, but the quadriceps strength was significantly lower for MKOA patients. Alzahrani [73] and Hubley-Kozey et al. [84] represented that the medial and lateral muscle cocontraction was increased for the KOA patients.

Knee ligament injury is a common and serious disease in sport injuries and can significantly change the biomechanics. According to where the injury hits, the knee ligament injury can be divided into the ACL, PCL, TCL, FCL, and PL injuries. Many researchers pointed out that the secondary injuries, e.g., cartilage injury, meniscus injury, and KOA, can occur if not treated in time. And the ligament reconstruction, as a recognized effective treatment, can dramatically recover the knee biomechanics [85–88]. In the five types of injures, nearly half of ligament injuries are isolated injuries to the ACL [89]. So, ACL injury will be mainly discussed in this section.

The biomechanical effects of ACL were shown in Table 13. In the knee kinematics aspect, Zhao et al. [90] reported that the knee ROM was lower for ACL-injured patients in stair climbing. Slater et al. [91] pointed out that the peak knee flexion angle was smaller and the peak knee adduction angle was greater for the ACL injury patients in walking. Cronstrom et al. [92] represented that the knee adduction degree during weight-bearing activities for ACL-injured patients was greater in walking. Gao and Zheng [93] indicated that the ACL-injured patients had slower speed and smaller stride length during walking. In the knee kinetics aspect, Alexander and Schwameder [94] observed a 430% and 475% increase in the patella-femur contact force for ACL-injured patients during upslope and downslope, respectively. Goerger et al. [95] found that the peak knee adduction moment during weight-bearing activities was greater in patients after ACL than before injury. Slater et al. [91] reported that a smaller peak external knee flexion and adduction moment can be found in the ACL-injured patients during walking. Thomas and Palmieri-Smith [96] illustrated no difference in the external knee adduction moment among individuals with ACL injury and those who are healthy. Norcross et al. [85] demonstrated that the ACL-injured patients had a greater knee energy adsorption during landing.

Meniscus injury, as a sport-induced injury, is common among athletes and general population [86, 89]. The meniscus-injured patients are often coupled with traumatic ACL injury and can increase the stress and reduce the stability of the knee joint during extension and flexion motions [89]. Many studies described that the secondary diseases, e.g., cartilage wear and KOA, can occur if not treated in time [87, 88, 97]. According to the injured degree, different treatments including conservative treatment, meniscus suture, and meniscectomy, can be selected.

To our knowledge, there are rare research that study the biomechanical effects of meniscus injury, as shown in Table 13. Magyar et al. [87] represented that the walking speed and knee ROM of meniscus-injured patients were significantly smaller, and the cadence, step length, duration of support, and double support phase of meniscus-injured patients were remarkably larger in walking. Zhou [86] indicated that the maximum flexion angle and maximum abduction-adduction angle between meniscus injury patients and healthy subjects have no apparent difference. The meniscus-injured patients had a larger minimum flexion angle and a smaller maximum internal-external rotation angles in walking. And the knee stressed area was smaller and the knee pressure was larger for the meniscus-injured patients in walking.

#### 3.2.2. Knee Neurological Disorders and Its Biomechanical Effects

SCI, one of the main causes of mobility disorders, affects around 0.25-0.5 million people every year around the world especially the young [98]. Approximately 43% of SCI patients turn out to have paraplegia and the number is increasing year by year [99]. The SCI patients are at an increasing risk of many secondary medical complications, including muscle atrophy, pressure ulcer, bone density reduction, and osteoporosis [100, 101]. Standing and walking, as the most prevalent desires of these patients, can stimulate blood circulation, ease muscle spasm, and increase the bone mineral density [98, 102]. Some evidences showed that the SCI patients can reduce the secondary medical complications risk and recover motion capabilities by standing or walking for several hours per day [98, 99, 102, 103]. The biomechanical effects of SCI were shown in Table 14. Barbeau et al. [104] pointed out that the knee ROM and peak knee-swing-flexion angle were lower, and peak knee moment was larger for SCI patients in walking. Desrosiers et al. [105] found that the knee power was lower for SCI patients in uphill and downhill walking. Pepin et al. [106] indicated that the SCI patients presented a longer flexed knee at good contact and maintain the longer flexion throughout the stance phase of walking.

Stroke, a common cerebrovascular disease, has a high mortality and disability rate [107, 108]. There are about 7.0 million stroke survivals in China and 6.6 million in the United States [109, 110]. Stroke is known as the cause of paralysis, loss of motor function, paresis-weakness of muscle, plegia-complete loss of muscle action, and muscle atrophy [34, 108, 109]. Impaired walking and sit-stand transition are the main reason that poststroke patients cannot live independently [107, 108]. And about 30% of poststroke patients have difficulty in ambulation without assistance [109]. Some evidences showed that 70% of poststroke patients can recover their walking capabilities by rehabilitation [108, 111]. The biomechanical effects of stroke were shown in Table 14. Sridar et al. [109] indicated that the kinematic and kinetic performance of the poststroke patients will degrade, such as reduced walking speed, quadriceps muscle moment, and quadriceps muscle power. Chen et al. [112] revealed that the poststroke patients had lower knee flexion in the swing phase of walking. Stanhope et al. [113] found that the poststroke patients can compensate their poor knee flexion in walking through faster speed. Marrocco et al. [114] reported a greater dynamic medical knee joint loading in stroke subjects in walking. However, the external knee adduction and flexion moments in walking were not significantly different between the stroke patients and healthy subjects. Novak et al. [115] observed that less energy was transferred concentrically via knee extensor muscles of stroke patients in mid-stance of walking. And the stroke patients presented lower energy absorption by the knee extensors in the late stance of walking.

CP, the most common pediatric neuromotor disorder, affects around 0.2-0.3% live births [19, 116]. The injury in the central nervous system of the developing fetus or infant is the pathogenesis of CP, which effects the control of movement, balance, and posture [116, 117]. The person with CP always has a variety of characteristics including rigidity, spasticity, abnormal aerobic and anaerobic capacity, decreased muscle strength and endurance, abnormal muscle tone, deformities, and muscle weakness [19, 118, 119]. The biomechanical effects of CP were shown in Table 14. Crouch gait, characterized by excessive knee flexion in stance phase, is a frequent gait deviation in CP patients [19, 117, 118]. Hicks et al. [120] reported the minimum knee flexion angle during the stance phase exceed 40 deg for the CP patients. Compared with the normal gait, crouch gait is inefficient and consumes much more energy [19, 116]. For maintaining the excessive knee flexion posture in walking, the stress of the knee and surrounding muscles are increasing, which can lead to bony deformities, degenerative arthritis, joint pain, and patellar stress fractures and then result in the severity of crouch gait [19, 118, 121]. Some evidences showed that the mobility function can be preserved and the complications can be reduced by limiting excessive knee flexion in walking [118].

## 4. Discussion and Conclusions

Knee disorders, including musculoskeletal and neurological disorders, have serious influences on knee biomechanics. A number of researches related with the biomechanics of normal and diseased knee joint have been done during the last decades. Many advances have been made to understand the kinematics and kinetics of normal and diseased knee during different common motions. In the aspect of normal knee biomechanics, there is no clear assessment at the current state-of-the-art. The difference between the results of different researches is significant. In the aspect of diseased knee biomechanics, a lower knee flexion angle, walking speed, muscles strength, and a higher knee contact pressure were always observed. Understanding how pathologies affect the knee joint biomechanics is important for designing knee assistive devices and optimizing rehabilitation exercise program. However, the current understanding still has not met the requirement of a designer and rehabilitative physician. And it is hard to find a research that can systematic study all aspects of knee biomechanics completely. Thus, deeper understanding of the biomechanics of normal and diseased knee joint will still be an urgent need in the future.

Some limitations of the current studies must be noted. First, the current understanding on the knee biomechanics is not enough. Many research about the theoretical analysis of knee biomechanics are based on the mathematical modeling. Whether a link model or a simulation model, there is a difference between the model and the reality. And some simplification should always be made, such as the mechanical property, geometry, and relative motion of the bone, muscle, cartilage, etc. Thus, the current computational knee biomechanics cannot describe the real knee biomechanics completely. Second, the kinematics and kinetics results from different research are vastly different. The results are hard to apply in the designing knee assistive devices and optimizing rehabilitation exercise program directly. Therefore, the kinematics and kinetics analyses must be redone in actual use. Third, the studies about the biomechanical influences of knee disorders are mainly concentrated in walking. Little research has been done on other daily life activities, such as running, stair climbing, and sit-to-stand. Fourth, there is an insufficient recognition of the influence of disorders on the knee biomechanics. The influence will always be obtained by patients-normal comparative experiments. And there is severe shortage of deeper rational analyses of the influence.

There are several limitations of our review. First, only articles published in English were included posing a language bias to article selection. Second, the review findings are limited to the articles identified by the set search strategy. Third, the quality of evidence for each study was very low because of the study designs and high heterogeneity.

In the future, the biomechanics of the normal and diseased knee joint will constitute a key research direction. More realistic biomechanical models and computing methods will be further developed for a deeper understanding of the kinematics and kinetics of the knee joint. And more rational analyses about the biomechanical influences of knee disorders will be further established to design better assistive mechanisms.

## Figures and Tables

**Table 1:** Overview over the experimental results of knee angle for normal walking.

Study	Subjects (mean height ± SD (m), mean weight ± SD (kg))	Speed (m/s)	A (°)	B (°)	C (°)	D (°)	C-A (°)	ROM (°)
Collins et al. [125]	9 (1.84 ± 0.10, 77.4 ± 9.2)	1.25	12	3	61	-5	49	66
Zheng [21]	1 (1.78, 70)	1.2	22	10	66	6	44	60
Wang [126], Lee et al. [17]	1 (1.69, 63.5)	1.5	7	3	53	-2	46	55
Mooney and Herr [22]	6 (1.83 ± 0.06, 89 ± 8)	1.4	28	5	78	4	50	74
Shamaei et al. [3, 127]	3 (1.76 ± 0.75, 68.6 ± 2.2)	1.25	26	15	69	6	43	63
Blazkiewicz [128]	1 (1.85, 80)	—	10	-2	53	0	43	55
Sridar et al. [34]	3 (1.70 ± 0.05, 74.7 ± 8.4)	1.0	22	11	62	2	40	60
Knaepen et al. [129]	10 (1.82 ± 0.10, 77.5 ± 11.7)	0.69	14	8	62	8	48	54
Shirota et al. [130]	4 (1.80 ± 0.08, 74 ± 6.8)	1.24	13	5	65	1	52	64
Gordon et al. [131]	3 (1.80 ± 0.01, 96 ± 9)	1.0	6	0	55	16	49	55
Ding et al. [132]	8 (1.76 ± 0.06, 78.5 ± 9.9)	1.25	24	7	75	0	51	75
Winter [133], Li et al. [134]	1 (—, 58)	1.3	16	5	67	-2	51	69
Beyl et al. [135]	—	—	22	8	64	1	42	63
Baliunas et al. [136]	15 (1.68 ± 0.12, 74 ± 16)	0.98	17	3	60	-2	43	62
Yang et al. [137]	1 (1.75, 70)	1.0	14	3	56	6	42	53

A: first peak knee flexion angle; B: first peak knee extension angle; C: second peak knee flexion angle; D: second peak knee extension angle.

**Table 2:** Overview over the experimental results of knee moment for normal walking.

Study	Subjects (mean height ± SD (m), mean weight ± SD(kg))	Speed (m/s)	E (Nm/kg)	F (Nm/kg)	G (Nm/kg)	H (Nm/kg)	E-G (Nm/kg)	F-H (Nm/kg)	Range (Nm/kg)
Collins et al. [125]	9 (1.84 ± 0.10, 77.4 ± 9.2)	1.25	0.556	-0.245	0.207	-0.388	0.349	0.143	0.944
Shamaei et al. [3]	3 (1.76 ± 0.75, 68.6 ± 2.2)	1.25	0.335	-0.248	0.102	-0.379	0.233	0.131	0.714
Zheng [21]	1 (1.78, 70)	1.2	0.571	-0.171	0.114	0.086	0.457	-0.257	0.742
Mooney and Herr [22]	6 (1.83 ± 0.06, 89 ± 8)	1.4	0.766	-0.344	0.189	-0.378	0.577	0.034	1.144
Blazkiewicz [128]	1 (1.85, 80)	—	0.263	-0.675	0.225	-0.063	0.038	-0.612	0.938
Ding et al. [132]	8 (1.76 ± 0.06, 78.5 ± 9.9)	1.25	0.777	-0.204	0.204	-0.420	0.573	0.198	1.197
Winter [133], Li et al. [134]	1 (—, 58)	1.3	0.517	-0.155	0.189	-0.224	0.328	0.069	0.741
Yang et al. [137]	1 (1.75, 70)	1.0	0.129	-0.329	0.101	-0.257	0.028	-0.072	0.458
Dijk et al. [138]	8 (1.79 ± 0.04, 75.1 ± 6.5)	1.11	0.945	0.067	0.466	-0.320	0.479	-0.387	1.265
Briggs et al. [51]	20 (1.67 ± 0.11, 58.0 ± 12.6)	—	0.534	-0.276	0.190	—	0.344	—	—

E: first peak knee extension moment; F: first peak knee flexion moment; G: second peak knee extension moment; H: second peak knee flexion moment.

**Table 3:** Overview over the experimental results of knee power for normal walking.

Study	Subjects (mean height ± SD (m), mean weight ± SD (kg))	Speed (m/s)	I (W/kg)	J (W/kg)	K (W/kg)	L (W/kg)	Range (W/kg)
Collins et al. [125]	9 (1.84 ± 0.10, 77.4 ± 9.2)	1.25	-0.571	0.286	-1.057	-1.457	1.743
Zheng [21]	1 (1.78, 70)	1.2	-0.489	0.591	-0.469	-0.321	1.080
Mooney et al. [22]	6 (1.83 ± 0.06, 89 ± 8)	1.4	-0.889	0.834	-1.334	-1.639	2.473
Malcolm et al. [16]	8 (1.67 ± 0.02, 60 ± 1)	1.38	-1.736	0.502	-0.763	-2.712	3.214
Ding et al. [132]	8 (1.76 ± 0.06, 78.5 ± 9.9)	1.25	-0.968	0.606	-1.290	-1.677	2.283
Winter [133], Li et al. [134]	1 (—, 58)	1.3	-0.755	0.324	-0.924	-1.247	1.571
Yang et al. [137]	1 (1.75, 70)	1.0	-0.116	0.296	-0.403	-0.739	1.035
Dijk et al. [138]	8 (1.79 ± 0.04, 75.1 ± 6.5)	1.1	-1.242	0.586	-1.509	-1.329	2.095
Walsh et al. [139]	1 (—, 60)	0.8	-0.828	0.667	-1.935	-1.410	2.602

I: first peak knee absorption power; J: first peak knee generation power; K: second peak knee absorption power; L: third peak knee absorption power.

**Table 4:** Overview over the experimental results of knee angle for running.

Study	Subjects (mean height ± SD (m), mean weight ± SD (kg))	Speed (m/s)	A (°)	B (°)	C (°)	D (°)	C-A (°)	ROM (°)
Zheng [21]	1 (1.78, 70)	2.1	36	22	80	20	44	60
		2.8	49	20	90	17	41	73

Hamner and Delp [23]	10 (1.77 ± 0.04, 70.9 ± 7.0)	2.0	42	18	85	11	43	74
		3.0	44	16	103	12	59	91
		4.0	46	15	119	13	73	106
		5.0	47	15	129	14	82	115

Dollar et and Herr[122]	1 (—, 85)	3.2	43	23	89	21	46	68

Elliott [123]	6 (1.81 ± 0.08, 69 ± 11)	3.5	44	15	105	13	61	92

Sobhani et al. [140]	16 (1.77 ± 0.09, 69.8 ± 11)	2.48	48	17	86	10	38	76

Miller et al. [141]	12 (1.66 ± 0.05, 61 ± 4.7)	3.8	60	29	96	16	36	80

Ferber et al. [52]	20 (1.81 ± 0.06, 82.3 ± 11.8)	3.65	46	13	—	—	—	—

A: first peak knee flexion angle; B: first peak knee extension angle; C: second peak knee flexion angle; D: second peak knee extension angle.

**Table 5:** Overview over the experimental results of knee moment for running.

Study	Subjects (mean height ± SD (m), mean weight ± SD (kg))	Speed (m/s)	E (Nm/kg)	F (Nm/kg)	G (Nm/kg)	H (Nm/kg)	E-G (Nm/kg)	F-H (Nm/kg)	Range (Nm/kg)
Zheng [21]	1 (1.78, 70)	2.1	1.157	-0.030	0.274	-0.277	0.883	0.247	1.434
		2.8	1.749	0.320	0.320	-0.351	1.429	0.671	2.100

Hamner and Delp [23]	10 (1.77 ± 0.04, 70.9 ± 7.0)	2.0	1.798	-0.205	0.135	-0.697	1.663	0.492	2.495
		3.0	2.159	-0.226	0.269	-0.925	1.890	0.699	3.084
		4.0	2.402	-0.233	0.405	-1.147	1.997	0.914	3.549
		5.0	2.430	-0.259	0.585	-1.474	1.845	1.215	3.904

Dollar and Herr [122]	1 (—, 85)	3.2	1.571	0.175	0.175	-0.591	1.396	0.766	2.162

Elliott [123]	6 (1.81 ± 0.08, 69 ± 11)	3.5	2.196	-0.249	0.248	-0.775	1.948	0.526	2.971

Sobhani et al. [140]	16 (1.77 ± 0.09, 69.8 ± 11)	2.48	2.574	-0.221	0.307	-0.649	2.267	0.428	3.223

E: first peak knee extension moment; F: first peak knee flexion moment; G: second peak knee extension moment; H: second peak knee flexion moment.

**Table 6:** Overview over the experimental results of knee power for running.

Study	Subjects (mean height ± SD (m), mean weight ± SD (kg))	Speed (m/s)	I (W/kg)	J (W/kg)	K (W/kg)	L (W/kg)	Range (W/kg)
Zheng [21]	1 (1.78, 70)	2.1	-5.859	4.336	-2.231	-3.521	10.195
		2.8	-8.008	7.386	-3.456	-3.456	15.394
Dollar and Herr [122]	1 (—, 85)	3.2	-1.706	4.766	-1.525	-3.958	8.724
Elliott [123]	6 (1.81 ± 0.08, 69 ± 11)	3.5	-9.013	4.539	-2.439	-6.732	13.552
Sobhani et al. [140]	16 (1.77 ± 0.09, 69.8 ± 11)	2.48	-12.567	9.405	-2.371	-4.473	21.972
Ferber et al. [52]	20 (1.81 ± 0.06, 82.3 ± 11.8)	3.65	-5.462	2.739	—	—	—
Heiderscheit et al. [62]	45 (1.76 ± 0.10, 69.5 ± 13.1)	2.9	-6.948	5.422	—	—	—

I: first peak knee absorption power; J: first peak knee generation power; K: second peak knee absorption power; L: third peak knee absorption power.

**Table 7:** Overview over the experimental results of knee angle for stair ascent and stair descent.

Study	Subjects (mean height ± SD (m), mean weight ± SD (kg))	Riser × tread (cm × cm)	Type	A (°)	B (°)	ROM (°)
Riener et al. [24], Joudzadeh et al. [25]	10 (1.79 ± 0.05, 82.2 ± 8.5)	13.8 × 31.0	Ascent	91	9	82
			Descent	89	13	76
		17.0 × 29.0	Ascent	95	9	86
			Descent	93	15	78
		22.5 × 25.0	Ascent	102	10	92
			Descent	102	13	89

Mcfadyen and Winter [142]	3 (—, —)	22.0 × 28.0	Ascent	99	11	88
			Descent	105	19	86

Zhang et al. [143]	10 (1.74 ± 0.05, 72.7 ± 8.6)	18.0 × 28.0	Ascent	89	7	82
			Descent	96	10	86

Musselman [27]	17 (1.85 ± 0.12, 82 ± 14)	15.0 × 26.0	Ascent	83	5	78
			Descent	83	6	77

Protopapadaki et al. [144]	33 (1.69 ± 0.08, 67.5 ± 12.1)	18.0 × 28.5	Ascent	94	0	94
			Descent	91	1	90

Law [26]	19 (1.64 ± 0.08, 59.5 ± 7.8)	17.0 × 28.0	Ascent	95	11	84
			Descent	93	3	90

A: peak knee flexion angle; B: peak knee extension angle.

**Table 8:** Overview over the experimental results of knee moment for stair ascent and stair descent.

Study	Subjects (mean height ± SD (m), mean weight ± SD (kg))	Riser × tread (cm × cm)	Type	E (Nm/kg)	F (Nm/kg)	G (Nm/kg)	H (Nm/kg)	E-G (Nm/kg)	F-H (Nm/kg)	Range (Nm/kg)
Riener et al. [24] and Joudzadeh et al. [25]	10 (1.79 ± 0.05, 82.2 ± 8.5)	13.8 × 31.0	Ascent	1.055	-0.179	0.027	-0.183	1.028	0.004	1.238
			Descent	0.916	0.587	1.247	-0.096	-0.331	0.683	1.343
		17.0 × 29.0	Ascent	1.093	-0.218	0.042	-0.177	1.051	-0.041	1.311
			Descent	1.006	0.662	1.345	-0.091	-0.339	0.753	1.436
		22.5 × 25.0	Ascent	1.164	-0.247	0.037	-0.172	1.127	0.075	1.411
			Descent	0.991	0.653	1.470	-0.088	-0.479	0.741	1.558

Mcfadyen and Winter[142]	3 (—, —)	22.0 × 28.0	Ascent	1.409	-0.406	0.164	-0.314	1.245	-0.092	1.815
			Descent	1.512	0.405	1.620	-0.266	-0.108	0.671	1.886

Zhang et al. [143]	10 (1.74 ± 0.05, 72.7 ± 8.6)	18.0 × 28.0	Ascent	0.588	-0.493	0.144	-0.256	0.444	-0.237	1.081
			Descent	0.338	0.152	1.106	-0.201	-0.768	0.353	1.307

Musselman [27]	17 (1.85 ± 0.12, 82 ± 14)	15.0 × 26.0	Ascent	0.921	-0.456	0.043	-0.206	0.878	-0.250	1.377
			Descent	0.448	0.263	1.012	-0.167	-0.564	0.430	1.179

Protopapadaki et al. [144]	33 (1.69 ± 0.08, 67.5 ± 12.1)	18.0 × 28.5	Ascent	0.454	-0.556	0.032	-0.121	0.422	-0.435	1.010
			Descent	0.007	-0.070	0.365	-0.040	-0.358	-0.030	0.435

Law [26]	19 (1.64 ± 0.08, 59.5 ± 7.8)	17.0 × 28.0	Ascent	0.899	-0.145	0.046	-0.147	0.085	0.002	1.036
			Descent	0.603	0.439	1.006	-0.076	-0.403	0.515	1.082

E: first peak knee extension moment; F: first peak knee flexion moment; G: second peak knee extension moment; H: second peak knee flexion moment.

**Table 9:** Overview over the experimental results of knee power for stair ascent and stair descent.

Study	Subjects (mean height ± SD (m), mean weight ± SD (kg))	Riser × tread (cm × cm)	Type	I (W/kg)	J (W/kg)	K (W/kg)	L (W/kg)	Range (W/kg0
Riener et al. [24] and Joudzadeh et al. [25]	10 (1.79 ± 0.05, 82.2 ± 8.5)	13.8 × 31.0	Ascent	2.322	0.071	0.647	-0.309	2.631
			Descent	0.256	-0.678	-0.429	-3.788	4.044
		17.0 × 29.0	Ascent	2.538	0.055	0.696	-0.312	2.850
			Descent	0.305	-1.029	-0.453	-4.141	4.446
		22.5 × 25.0	Ascent	2.887	0.049	0.811	-0.288	3.175
			Descent	-0.212	-1.255	-0.472	-4.843	4.631

Mcfadyen and Winter [142]	3 (—, —)	22.0 × 28.0	Ascent	2.742	-0.228	1.020	-0.739	3.481
			Descent	0.569	-3.621	-1.326	-5.485	6.054

Musselman [27]	17 (1.85 ± 0.12, 82 ± 14)	15.0 × 26.0	Ascent	1.044	-0.223	0.447	-0.265	1.309
			Descent	0.037	-0.248	-0.558	-2.077	2.114

I: first peak knee generation power; J: first peak knee absorption power; K: second peak knee generation power; L: second peak knee absorption power.

**Table 10:** Overview over the experimental results of knee angle for sit-to-stand.

Study	Subjects (mean height ± SD (m), mean weight ± SD (kg))	A (°)	B (°)	ROM (°)
Wu et al. [37]	1 (—, 75)	96	9	87
Hurley et al. [28]	10 (1.77 ± 0.08, 77 ± 13)	90	12	78
Spyropoulos et al. [29]	17 (1.65 ± 0.07, 54.6 ± 5)	86	-1	87
Karavas et al. [124]	1 (1.85, 82.5)	86	5	81
Yu et al. [145]	10 (1.65 ± 0.05, 46.2 ± 0.8)	82	22	60
Bowser et al. [146]	12 (1.66 ± 0.08, 74.2 ± 19.5)	83	-3	86

A: peak knee flexion angle; B: peak knee extension angle.

**Table 11:** Overview over the experimental results of knee moment for running.

Study	Subjects (mean height ± SD (m), mean weight ± SD (kg))	E (Nm/kg)	F (Nm/kg)	Range (Nm/kg)
Wu et al. [37]	1 (—, 75)	2.187	0.609	1.578
Hurley et al. [28]	10 (1.77 ± 0.08, 77 ± 13)	0.619	0	0.619
Yoshioka et al. [147]	1 (—, 73.8)	1.087	-0.038	1.125
Spyropoulos et al. [29]	17 (1.65 ± 0.07, 54.6 ± 5)	1.132	-0.157	1.289
Karavas et al. [124]	1 (1.85, 82.5)	1.293	0.168	1.125
Bowser et al. [146]	12 (1.66 ± 0.08, 74.2 ± 19.5)	0.901	0	0.901
Kamali et al. [30]	1 (1.72, 70)	1.126	0.136	0.990
Schofield et al. [148]	10 (1.77 ± 0.09, 70.5 ± 8.7)	0.679	0.038	0.641
Robert et al. [149]	7 (1.75 ± 0.06, 66 ± 8)	1.136	-0.198	1.334

E: peak knee extension moment; F: peak knee flexion moment.

**Table 12:** Overview over the biomechanical effects of KOA.

Study	Analysis	Effects
Chao et al. [70]	Medical radiology	HKAA: ~178.8 deg for normal knee; <178 deg for MKOA patients

Paley [71]	Medical radiology	mLDFA: 85-90 deg for normal knee; >90 deg for MKOA patients MPTA: 85-90 deg for normal knee; <85 deg for MKOA patients JLCA: 0-2 deg for normal knee; >2 deg for MKOA patients MAD: ~8 mm for normal knee; >8 mm for MKOA patients

Russell [50]	Medical radiology	HKAA: ~177.7 deg for normal knee; ~174.2 deg for MKOA patients WBL ratio: ~41.4% for normal knee; ~24.2% for MKOA patients Medial joint apace: ~4.5 mm for normal knee; ~2.8 mm for MKOA patients Lateral joint apace: ~5.5 mm for normal knee; ~7.9 mm for MKOA patients
	Kinematics	A lower knee flexion angle for MKOA patients
	Kinetics	A higher knee adduction moment for MKOA patients
	Muscles	A lower quadriceps strength for MKOA patients

Zhu et al. [72]	Kinematics	A longer gait time, a smaller stride length and ROM, a greater knee flexion angle at heel strike, and an unobvious fluctuation of knee flexion angle in stand phase of walking for MKOA patients

Alzahrani [73]	Kinematics	A slower walking speed, a shorter step length, a longer stance, and double support time, and smaller cadence, stride length, and knee ROM for MKOA patients
	Muscles	The medial and lateral muscle cocontraction was increased for KOA patients

Astephen et al. [74]	Kinetics	A greater knee adduction moment in mid-stance for MKOA patients

Guo et al. [75]	Kinetics	A greater peak adduction moment during stair climbing for MKOA patients

Rudolph et al. [76] and Schmitt and Rudolph [77]	Kinetics	A smaller peak knee flexion moment during early and late stance phases for MKOA patients

Fitzgerald [78]	Kinetics	A 4-6 deg increase in varus alignment could increase around 70-90% medial compartment load during single limb bearing

Lim et al. [79]	Kinetics	Genu varum exceeding 5 deg was associated with greater functional deterioration over 18 months than the value of 5 deg or less
	Muscles	No significant relationship between the varus malalignment and the EMG ratio of VM and VL

Kemp et al. [80]	Kinetics	A 20% increase in the peak adduction moment could increase the KOA progression risk

Slemenda et al. [81], Hurley et al. [82], and Oreilly et al. [83]	Muscles	A smaller quadriceps strength and muscle activation for KOA patients

Hubley-Kozey et al. [84]	Muscles	The medial and lateral muscle cocontraction was increased for KOA patients

**Table 13:** Overview over the biomechanical effects of ACL and meniscus injury.

Study	Knee disorders	Analysis	Effects
Zhao et al. [90]	ACL	Kinematics	A lower knee ROM during stair climbing for ACL-injured patients

Gronstrom et al. [92]	ACL	Kinematics	A greater knee adduction angle during weight-bearing activities for ACL-injured patients

Gao and Zheng[93]	ACL	Kinematics	A slower speed and smaller stride length during walking for ACL-injured patients

Alexander and Schwameder[94]	ACL	Kinetics	A 430% and 475% increase in the patella-femur contact force during upslope and downslope, respectively, for ACL-injured patients.

Goerger et al. [95]	ACL	Kinetics	A greater peak knee adduction moment during weight-bearing activities for ACL-injured patients

Slater et al. [91]	ACL	Kinematics	A smaller peak knee flexion angle and a greater peak knee adduction angle during walking for ACL-injured patients
		Kinetics	A smaller peak E-KFM and E-KAM for ACL-injured patients

Thomas et al. [96]	ACL	Kinetics	No difference in the E-KAM among individuals with ACL injury and those who are healthy

Norcross et al. [85]	ACL	Kinetics	A greater knee energy adsorption for ACL-injured patients

Magyar et al. [87]	Meniscus injury	Kinematics	A smaller walking speed and knee ROM and a larger cadence, step length, duration of support, and double support phase for meniscus injured patients

Zhou [86]	Meniscus injury	Kinematics	A larger minimum flexion angle and a smaller maximum internal-external rotation angle for meniscus-injured patients
		Kinetics	A larger knee pressure and a smaller knee stressed area for meniscus-injured patients

**Table 14:** Overview over the biomechanical effects of SCI, stroke, and CP.

Study	Knee disorders	Analysis	Effects
Barbeau et al. [102]	SCI	Kinematics	A lower knee ROM and peak knee-swing-flexion angle for SCI patients
		Kinetics	A larger peak knee moment for SCI patients

Desrosiers et al. [103]	SCI	Kinetics	A lower knee power during uphill and downhill walking for SCI patients

Pepin et al. [104]	SCI	Kinematics	A longer knee flexion at good contact and maintain the longer flexion throughout the stance phase of walking for SCI patients.

Sridar et al. [109]	Stroke	Kinematics	A lower walking speed for stroke patients
		Muscles	A lower quadriceps muscle moment and power for stroke patients

Chen et al. [112]	Stroke	Kinematics	A lower knee flexion in the swing phase of walking for poststroke patients

Stanhope et al. [113]	Stroke	Kinematics	Post-stroke patients can compensate their poor knee flexion in walking through faster speed

Marrocco et al. [114]	Stroke	Kinetics	A greater dynamic knee joint loading for stroke patients and no significant difference between the E-KFM/E-KAM of stroke and healthy subjects.

Novak et al. [115]	Stroke	Kinetics	A less energy transference in mid-stance of walking and a lower energy absorption in the late stance of walking for stroke patients

Lerner [19] and Thapa et al. [116]	CP	Kinetics	Crouch gait (characterized by excessive knee flexion in stance phase), walking inefficiency, and consumes much more energy

Hicks et al. [120]	CP	Kinematics	Minimum knee flexion angle during the stance phase exceeding 40 deg for CP patients
